# Screening adequacy of unstained thyroid fine needle aspiration samples using a deep learning-based classifier

**DOI:** 10.1038/s41598-023-40652-1

**Published:** 2023-08-19

**Authors:** Junbong Jang, Young H. Kim, Brian Westgate, Yang Zong, Caleb Hallinan, Ali Akalin, Kwonmoo Lee

**Affiliations:** 1 Department of Biomedical Engineering, Worcester Polytechnic Institute, Worcester, MA 01609 USA; 2 Vascular Biology Program, Boston Children’s Hospital, Boston, MA 02115 USA; 3 Department of Radiology, University of Massachusetts Medical School, Worcester, MA 01655 USA; 4 Department of Pathology, University of Massachusetts Medical School, Worcester, MA 01655 USA; 5 Department of Surgery, Harvard Medical School, Boston, MA 02115 USA

**Keywords:** Image processing, Machine learning, Biomedical engineering, Medical imaging

## Abstract

Fine needle aspiration (FNA) biopsy of thyroid nodules is a safe, cost-effective, and accurate diagnostic method for detecting thyroid cancer. However, about 10% of initial FNA biopsy samples from patients are non-diagnostic and require repeated FNA, which delays the diagnosis and appropriate care. On-site evaluation of the FNA sample can be performed to filter out non-diagnostic FNA samples. Unfortunately, it involves a time-consuming staining process, and a cytopathologist has to be present at the time of FNA. To bypass the staining process and expert interpretation of FNA specimens at the clinics, we developed a deep learning-based ensemble model termed FNA-Net that allows in situ screening of adequacy of unstained thyroid FNA samples smeared on a glass slide which can decrease the non-diagnostic rate in thyroid FNA. FNA-Net combines two deep learning models, a patch-based whole slide image classifier and Faster R-CNN, to detect follicular clusters with high precision. Then, FNA-Net classifies sample slides to be non-diagnostic if the total number of detected follicular clusters is less than a predetermined threshold. With bootstrapped sampling, FNA-Net achieved a 0.81 F1 score and 0.84 AUC in the precision-recall curve for detecting the non-diagnostic slides whose follicular clusters are less than six. We expect that FNA-Net can dramatically reduce the diagnostic cost associated with FNA biopsy and improve the quality of patient care.

## Introduction

Fine needle aspiration (FNA) biopsy of thyroid nodules is a safe, cost-effective, and the most accurate method for diagnosing whether the thyroid nodule is benign or cancerous^1^. It is estimated that about 300,000 new thyroid nodules occur annually in the United States^2^. However, about 10% of initial FNA biopsy samples are non-diagnostic (i.e., inconclusive results) due to several reasons: cystic fluid or bloody smears in the aspirated sample, operator experience, needle type, aspiration technique, vascularity of nodule, and the criteria used to judge the adequacy of the specimen^2,3^. The failure to obtain adequate biopsy samples poses a delay in the treatment of patients with thyroid cancer because the patient must return for another FNA biopsy. Considering a 5% malignancy rate in patients with initial non-diagnostic FNA^4^, some patients lose a precious opportunity to get treatments promptly.

In the current standard of care, interpretation of FNA results is performed in the cytology department well after the biopsy procedure is completed and biopsy samples are stained. Papanicolaou staining makes the nuclei of follicular cells dyed blue and dying cells or cell debris in red so that follicular cells are easily distinguished from other cells^5,6^. After staining, pathologists evaluate the adequacy of the FNA samples from patients and decide their adequacy. The adequacy of the unstained sample can be assessed by Rapid On-Site Evaluation (ROSE)^7,8^ or Rapid On-Site Adequacy Assessment (ROSAA)^9^ at the time of FNA at the clinics. However, ROSE or ROSAA needs trained cytopathologists with limited availability or alternative evaluators who are less accurate^10^. Even though staining is necessary for correct pathologic diagnosis, there is not a significant difference in the accuracy of assessing the adequacy between unstained and stained FNA samples smeared on slides^11^. Therefore, to avoid the additional cost and time involved in the staining procedures, we aim to screen the adequacy of the unstained FNA sample automatically by a computational pipeline without trained experts at the point of care.

Distinguishing follicular cells from other cells in the unstained FNA biopsy sample is challenging due to the irregular shapes and complex mixtures composed of follicular cells, red blood cells, lymphocytes, macrophages, endothelial cells, and stromal tissues. Despite this difficulty, a machine learning algorithm could learn morphological descriptors to detect red blood cells on an unstained slide^12^. Moreover, the deep learning-based model that uses Convolutional Neural Network (CNN) has superior performance to the traditional machine learning methods in the classification of ImageNet^13^. CNN is successfully applied to classify papillary thyroid carcinomas in stained FNA biopsy samples^14,15^ and predict malignancy in whole slide image of thyroid FNA samples^16^. However, to the best of our knowledge, the deep learning method has not been applied to screen the adequacy of unstained FNA samples.

We present the deep learning-based FNA whole slide classifier (FNA-Net) that can screen the adequacy of unstained FNA slides. FNA-Net comprises two models, patch-based whole slide image classifier^17^ and Faster R-CNN^18^. FNA-Net combines the follicular detection results from two models by overlapping them to make the final detection. The patch-based whole slide image classifier has the VGG19 encoder pretrained on ImageNet^19^, effective for a limited amount of data^20–23^. Moreover, the patch-based whole slide image classifier is trained by multi-task learning (MTL), in which auxiliary tasks are regression, image reconstruction and segmentation. Thus, we abbreviate the patch-based whole slide image classifier as the MTL classifier. MTL applied to deep learning improves the performance of classification^20^ and clustering^24,25^ by having the the shared encoder that learns the shared representation across different tasks. The previous study employed trainable weight parameters to balance one classification and two regression tasks by automatically tuning loss weights among different tasks^26^. We utilize this method to train MTL classifier without manually adjusting training loss weights for each task.

Finally, the follicular cluster detection results from every patch of the whole slide are used to classify the adequacy of the whole slide by counting the number of patches containing follicular clusters. For evaluation, we bootstrapped the test images, representing one slide, into 10,000 slides which are either adequate or inadequate based on the number of follicular clusters present in total. Under our simulated setting, FNA-Net achieved a 0.81 F1 score and 0.84 AUC in the precision-recall curve for detecting the non-diagnostic slides which contain less than six follicular clusters.

## Results

### FNA-net pipeline

**Figure 1:**
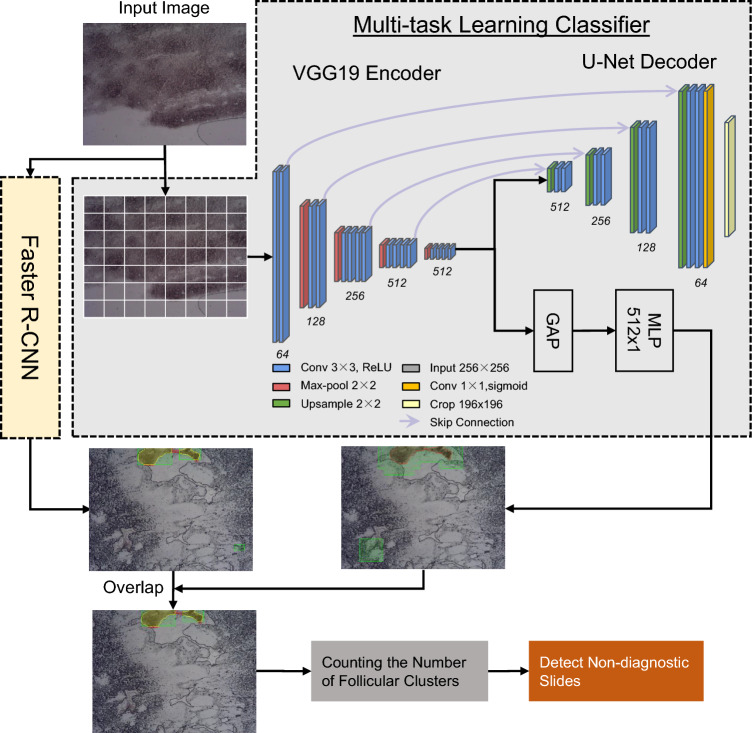
FNA-Net Architecture. Combination of detection results from Multi-task learning (MTL) classifier and Faster R-CNN that take the same FNA biopsy image as an input. MTL classifier crops the large image into 256 × 256 pixel patches before training or inference. ReLU activation layer comes after every convolutional layer marked by blue. GAP is a global average pooling layer that aggregates 256 × 256 × 512 feature maps into 1 × 1 × 512 feature vectors by averaging the spatial dimensions. The U-Net decoder performs segmentation as an auxiliary task during training to improve classification accuracy. Segmentation results from the U-Net decoder are not used in the inference step for follicular cluster detection. U-Net decoder has skip connections that concatenate encoded features from VGG19 Encoder with the decoded features. MLP layer outputs one value through sigmoid activation layer, indicating whether one 256 × 256 pixel patch contains a follicular cluster. All patches containing follicular clusters are stitched together to form one detection result on the unstained image. During inference, the follicular cluster detection results from the MTL classifier and Faster R-CNN are overlapped to finalize inference, the follicular cluster detection.

The FNA-Net pipeline works in two stages. In the first stage (Fig. 1), FNA-Net trains on the paired unstained and follicular cluster labeled images in a training set to detect follicular clusters with high precision. Two deep learning models, patch-based whole slide image classifier (MTL Classifier) and Faster R-CNN trains separately. Their follicular cluster detections are overlapped only for evaluation on the test set. In the second stage, the adequacy of the unstained FNA sample is classified based on the total number of detected follicular clusters from the first stage. We count all detected follicular clusters in all image patches from a slide and classify the slide as adequate if the number of predicted follicular is greater than or equal to the threshold predetermined by the user (user threshold). Only the first stage requires training and inference of the deep learning models, and the second stage performs simple algorithmic operations based on the results from the first stage.

### Training dataset

**Figure 2:**
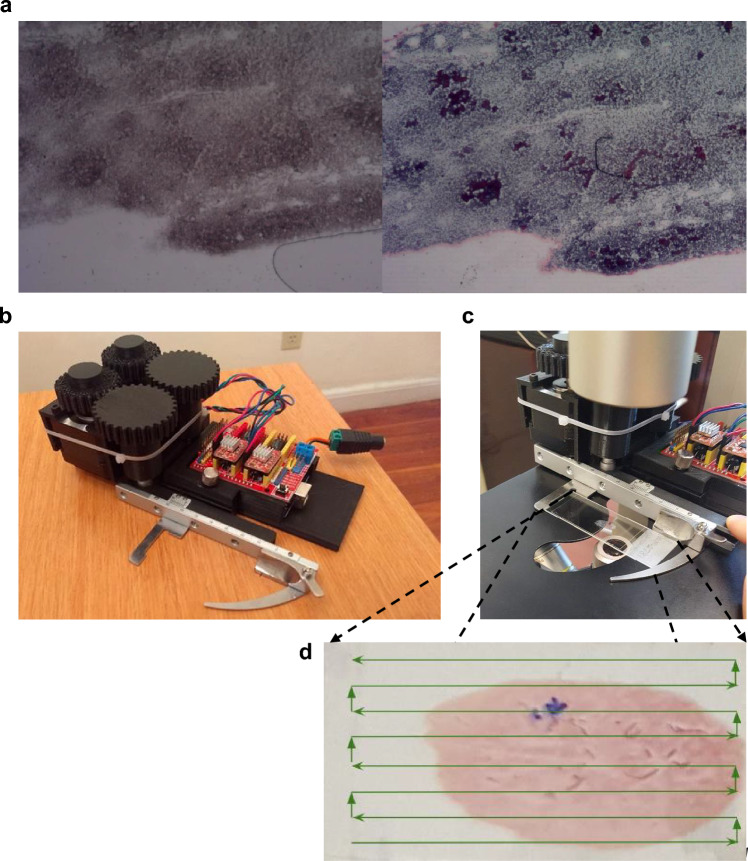
Unstained and Stained image of Fine Needle Aspiration sample. (**a**) Unstained image on the left and its corresponding stained image on the right are taken at 20X magnification. The stained image is approximately registered to the original image. The blue and purplish regions in the stained image indicate where follicular clusters are likely located. (**b**–**d**) Data Acquisition by Low-cost Slide Scanner. (**b**)Low-cost slide scanner comprised of gears, motors, electrical components such as Arduino and the glass slide clip. (**c**) Low-cost slide scanner attached to the bright-field microscope with the glass slide on the clip. (**d**) Thyroid Fine Needle Aspiration sample smeared on a glass slide is taken in order indicated by the green arrows.

A low-cost slide scanner (Fig. 2b) attached to a bright-field microscope was used to take images of all FNA biopsy samples (Fig. 2c, d). The unstained slides were imaged and then stained with Papanicolaou stain and reimaged (Fig. 2a). In total, unstained and stained FNA biopsy samples were taken from six patients, with 21 slides to evaluate (see Methods for details). Ground truth follicular clusters in the unstained images were labeled with the help of pathologists. Follicular cells tend to be clustered together, while other morphologically similar cells such as lymphocytes or macrophages tend to be dyscohesive or isolated from each other. Compact follicular cell clusters covered by blood or stromal tissues were not labeled since they are challenging to see through and use for diagnosis.

### Patch-wise follicular cluster classification with multi-task learning

We took a multi-task learning (MTL) approach for patch-wise classification by adding auxiliary tasks, which are regression, image reconstruction, and segmentation, to the classification model. For classification and regression tasks, one MLP layer with 512 hidden units was used. For segmentation and image reconstruction tasks, upsampling structure like U-Net^27^ decoder was used (Fig. 1). The classification task detects any follicular cluster in the image patch. The regression task estimates the size of the follicular clusters in pixels per image patch. The image reconstruction task reconstructs the input image as close as possible. The segmentation task predicts the follicular cluster’s location pixel-wise.

**Figure 3:**
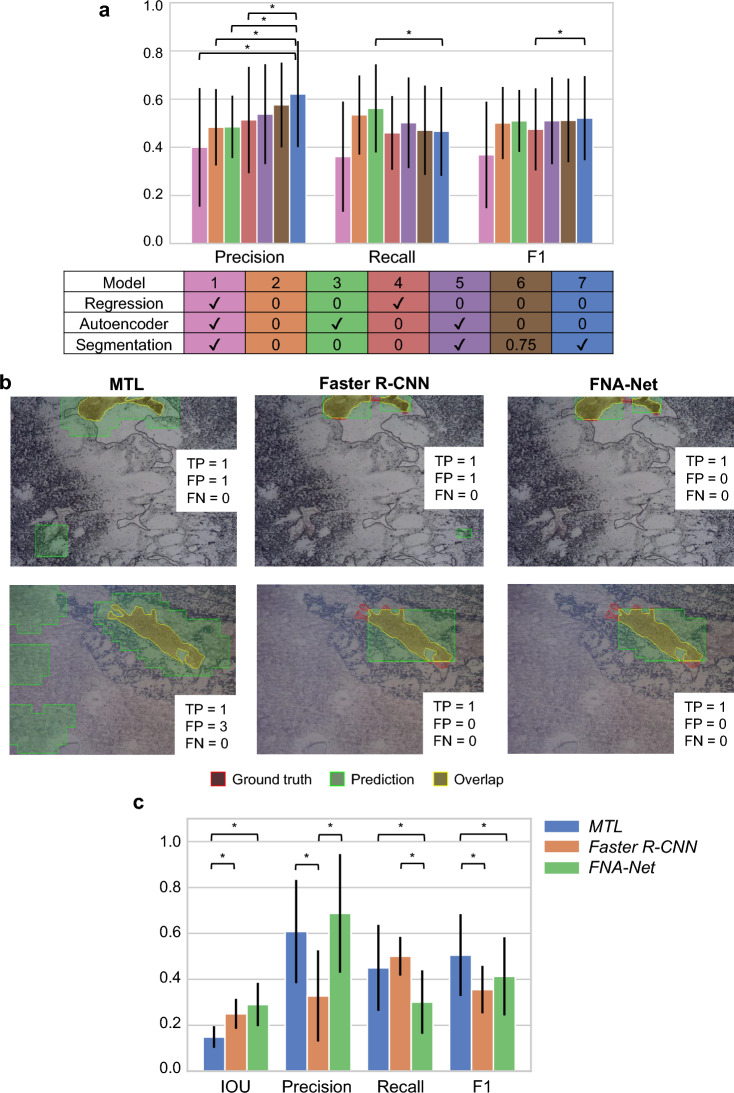
Follicular cluster detection results by MTL, Faster R-CNN, and FNA-Net. (**a**) Performance of multi-task learning models. The color of each model in the table corresponds to the color of bar in the bar graph. Weights for classification task is fixed to 1 and weights for other tasks are specified (✓: variable, the weights are automatically optimized during training). The statistical significance between the Model 7 and each of the other models are shown only. Significance was tested by the two-sided Wilcoxon signed-rank test. *p < 0.05. Error bars: one standard deviation. (**b**) The first column is the follicular cluster detection by the MTL model. The second column is the follicular cluster detection by the Faster R-CNN model. The third or last column is the follicular cluster detection by combing predictions from MTL and Faster R-CNN models. In the corner of each image, the number of true positives, false positives and false negatives are indicated next to the letters TP, FP, and FN, respectively. Each row has the same underlying image with the same ground truth. The ground truth is colored red, detection by the model is colored green, and the overlap between ground truth and detection is colored yellow. (**c**) The bar graph compares the average IOU, precision, recall, and F1 among MTL, Faster R-CNN, and FNA-Net. All four metrics range from 0 to 1, higher the better. The statistical significances between models within each metric (IOU, precision, recall, and F1 score) are shown. Significance was tested by the two-sided Wilcoxon signed-rank test. *p < 0.05. Error bars: one standard deviation.

The total loss is calculated by a weighted sum of classification, regression, image reconstruction (autoencoder), and segmentation losses. Since finding optimal loss weights manually is time-consuming and computationally expensive, we optimized the loss weights as trainable parameters during training^26^. Among combinations of tasks used to train MTL models, having segmentation tasks together with classification (Model 7) yielded the best performance in precision while autoencoder (Model 3) produced the highest recall (Fig. 3a, Tables 1, S1). In our application, high precision is preferred to high recall because overestimating the number of follicular cells incurs higher medical cost. Adding regression tasks to the MTL model (Model 1) was detrimental to the classification accuracy. Instead of having as many tasks as possible, which can lead to negative transfer^28^, choosing a few relevant tasks for the primary task yields better performance. Also, we showed that optimizing the loss weights during training yields higher performance than assigning equal weights to each loss or manually tuning loss weights given limited computational resources. Among the loss weights manually chosen for segmentation tasks such as 0.25, 0.5, 0.7, 0.75, 0.8, and 1, the weight 0.75 yielded the best classification accuracy (Model 6). However, this manually chosen loss weight did not outperform the loss weights optimized during training.

**Table 1:** Patch-wise classification performance of multi-task learning models.

Number of tasks	Task weights				Classification results		
	Class	Reg	Aut	Seg	Precision	Recall	F1
1	1	0	0	0	0.483	0.534	0.501
2	1	✓	0	0	0.513	0.460	0.474
2	1	0	✓	0	0.485	**0.562**	0.510
2	1	0	0	✓	**0.621**	0.466	**0.521**
2	1	0	0	0.75	0.576	0.471	0.512
3	1	0	✓	✓	0.538	0.502	0.510
4	1	✓	✓	✓	0.400	0.361	0.368

Weights for classification task is fixed to 1, and weights for other tasks are either 0 or ✓, which means that the weights are automatically optimized during training. The mean precision, recall and F1 from sevenfold cross validation are shown for the classification results. Reg, Aut and Seg represent regression, autoencoder, and segmentation tasks, respectively. The highest value for each metric is highlighted in bold.

### Follicular cluster detection by MTL, faster R-CNN, and FNA-net

For final follicular cluster detection, FNA-Net combines outputs from the best performing MTL model found in the previous section with results from separately trained Faster R-CNN. Similar to our MTL models, Faster R-CNN^18^ also has multiple tasks, which are classification and bounding box regression tasks in the architecture. However, Faster R-CNN is trained on the whole image without cropping it into smaller patches. Follicular cluster detections by MTL, Faster R-CNN, and FNA-Net (the intersection of MTL and Faster R-CNN) models on unstained images are shown in Fig. 3b and Supplementary Fig. 1. MTL detects large regions as follicular clusters, which overestimates the size of ground truth follicular clusters. In contrast, Faster R-CNN detects a much fewer number of follicular clusters that are smaller in size but misses the ground truth follicular clusters. When the detections from MTL and Faster R-CNN models are combined by overlapping them, the false detection from MTL is ignored by Faster R-CNN, and the false detection from Faster R-CNN is sometimes ignored by the MTL model. As a result, FNA-Net increases the IOU and reduces the number of false positives which increases the precision.

For evaluation, we counted the number of true positives (TPs), false positives (FPs) and false negatives (FNs). True positive means that one or more predicted follicular cluster boxes overlap with the ground truth box by at least one pixel (see Methods for details). There are 41 images in the test set containing 37 ground truth follicular clusters in total. The overlapped area is not considered in the evaluation because our goal is to count follicular clusters in the slide to assess its adequacy, instead of localizing the follicular clusters. For instance, in one of the test set, MTL model has 27 TPs, 10 FNs and 34 FPs. Faster R-CNN model has 16 TPs, 21 FNs and 6 FPs. FNA-Net has 16 TPs, 21 FNs and 3 FPs. FNA-Net reduced the number of FPs by half while having the same number of TPs and FNs as Faster R-CNN model.

Based on the number of TPs, FNs, and FPs, the precision, recall, and F1 scores are calculated (see Methods for details) (Fig. 3c, Table S1). FNA-Net achieved significantly higher average IOU than MTL. FNA-Net’s IOU, precision, recall, and F1 score are 0.290, 0.688, 0.305, and 0.417, respectively. MTL model’s IOU, precision, recall, and F1 score are 0.149, 0.621, 0.466, and 0.521, respectively. Faster R-CNN model’s IOU, precision, recall, and F1 score are 0.250, 0.329, 0.505, and 0.359, respectively. Interestingly, the MTL model has high precision and low recall, while the Faster R-CNN has high recall and low precision. FNA-Net significantly improved its precision and IOU over MTL (p-value: 0.0156) by combining two different models with opposite precision-recall performance. The precision of FNA-Net was significantly increased over Faster-RCNN (p-value: 0.0156) because FNA-Net mainly reduces the number of false positives, which affects the precision. The detection of follicular clusters with high IOU and precision is crucial for minimizing the overestimation of the number of follicular clusters in the sample slides as shown in the next section.

### Simulated screening adequacy of the slides by bootstrapping

The follicular cluster detection accuracy per image does not reflect the model’s ability to screen thyroid FNA slides. Instead, we need multiple slides in a test set to evaluate the screening accuracy. Due to the limited amount of testing set, multiple slides with and without adequate follicular clusters were simulated from the test set by bootstrapping, which is random sampling with replacement. Hierarchical bootstrapping was used instead of the normal bootstrapping because our dataset is multi-level, in which the first level corresponds to patients and the second level corresponds to the images from a patient’s FNA biopsy. In this case, hierarchical bootstrapping allows a more balanced sampling of follicular and background classes than normal bootstrapping. The ratio between the number of follicular and background classes is about 1:20 in normal bootstrapping, whereas, in hierarchical bootstrapping, the ratio becomes about 1:3.

**Figure 4:**
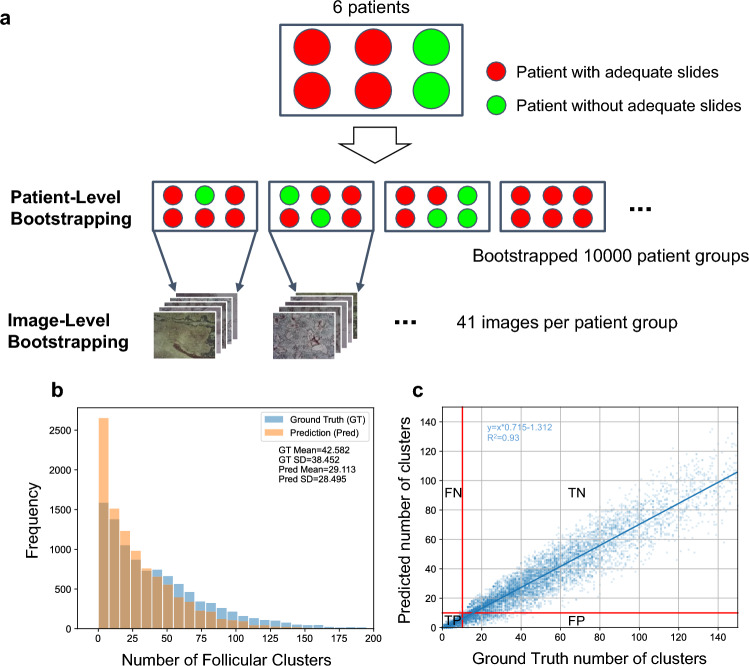
Hierarchical Bootstrapping Procedure and summary of the bootstrapped samples. (**a**) Hierarchical Bootstrapping. Any of six patients are randomly sampled with repetition to create 10,000 new groups by patient level bootstrapping. Then, 41 images are randomly sampled without repetition from six patients in a group by image level bootstrapping. The red circle represents a patient with an adequate FNA biopsy sample, and the green circle represents a patient who does not have an adequate FNA biopsy sample. One patient group containing six patients are bootstrapped patient-wise such that there are 10,000 patient groups with different combination of patients. (**b**) Distribution of samples of follicular clusters for ground truth and prediction. The orange color represents the bootstrapped predictions from the FNA-Net. The overlap between the ground truth in blue and the prediction in orange is shown in darker orange color. (**c**) Scatter plot of predicted and ground truth number of follicular clusters in the bootstrapped samples. Each blue dot represents a bootstrapped sample. The linear line in dark blue is fit to the scatter points. The linear line’s slope, y-intercept, and goodness of fit R^2^ are on the top of the graph. Red lines are drawn at threshold value equal to 10 which separate the graph into the four quadrants. All bootstrapped samples belong to one of four quadrants indicated by the two letters as follows; FP is false positive, TP is true positive, FN is false negative, and TN is true negative.

To generate new diversified datasets that retain the variability of sample adequacy and the variability of images from each patient, we performed the hierarchical bootstrapping comprised of patient-level and image-level bootstrapping (Fig. 4a). In the patient-level bootstrapping, six patients were randomly sampled with repetition 10,000 times, so that there are 10,000 bootstrapped patient groups comprised of six patients. Then, 41 testing images were sampled with repetition from each patient group to generate one bootstrapped slide. By repeating this step, 10,000 slides with either adequate or inadequate follicular clusters were bootstrapped. Each bootstrapped slide has 41 images with the corresponding number of follicular cluster detections and ground truth follicular clusters. The histograms in Fig. 4b show that the distribution of bootstrapped follicular cluster detection is more skewed to the left than the distribution of bootstrapped ground truth follicular clusters. This means that the bootstrapped test set contained fewer follicular cluster detections by the model than the number of ground truth follicular clusters because FNA-Net had relatively low recall values.

We define the threshold as the minimum number of follicular clusters to diagnose a slide to be adequate. There are two thresholds, the ground truth threshold determined by the medical experts and the user threshold that can be adjusted by the user depending on which metric, precision or recall, needs to be higher. The thyroid FNA sample smeared on the glass slide is evaluated to be adequate if the slide has at least six follicular clusters, each containing about 10–15 follicular cells^2,29,30^. Therefore, we set the ground truth threshold to 6. We also evaluated the ground truth threshold equal to 10, for the case where clinicians want to have a more stringent criterion for sample adequacy.

The scatter plot in Fig. 4c has the red line indicating the threshold value of 10 for both ground truth and the predicted minimum number of follicular clusters. These lines separate the plot into four quadrants. Starting from the top left quadrant and going clockwise, each quadrant represents false negative (FN), true negative (TN), false positive (FP), and true positive (TP), where the positive class is for inadequate (non-diagnostic) slides, and the negative class is for adequate (diagnostic) slides. Therefore, the true positive means that inadequate slide is correctly classified as inadequate (see Methods for details). The total number of points in each quadrant represents each of FN, TN, FP and TP. The number of TPs and TNs are the most abundant, and the number of FNs is the lowest, suggesting that it is much less likely to classify inadequate slides to be adequate.

**Figure 5:**
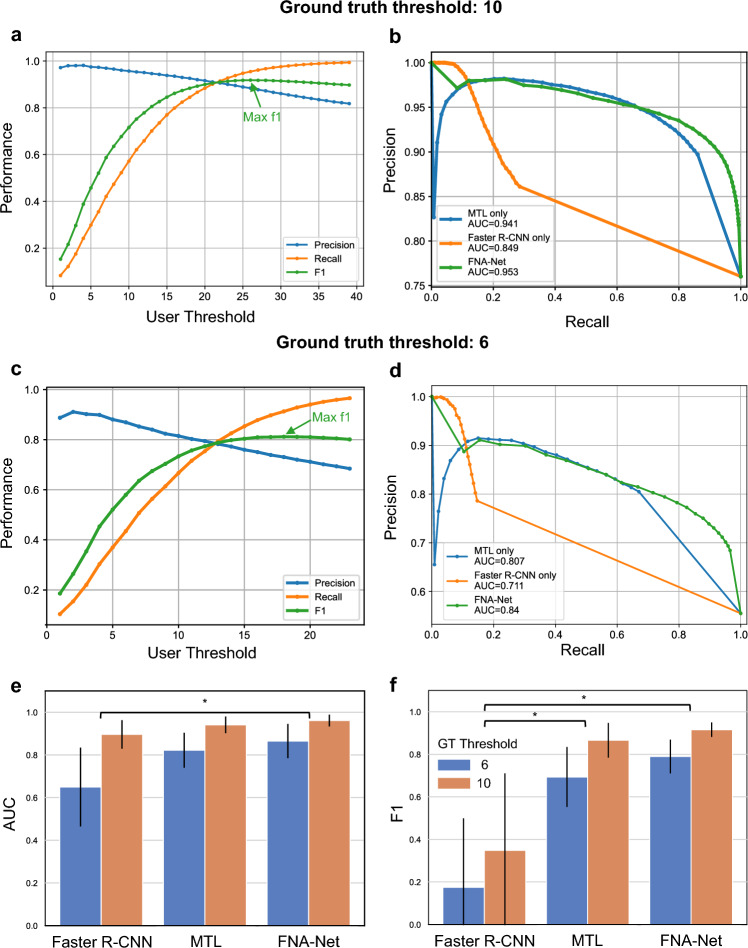
Evaluation of models’ screening performance on bootstrapped samples. (**a**, **c**) The F1, Precision, and Recall at user threshold values ranging from 1 to 39 to classify the slide as adequate. The user threshold is the minimum number of predicted follicular clusters. Precision, recall and F1 are indicated by blue, orange and green colorrespectively. Maximum F1 is indicated by green arrow. (**a**) Ground truth threshold = 10, (**c**) ground truth threshold = 6 (**b**, **d**) The Precision-Recall curves of the three follicular cluster detection models in classifying the adequacy of the slide. MTL, Faster R-CNN, and FNA-Net models are indicated by blue, orange, and green curves, respectively. The red line is the baseline model. (**b**) ground truth threshold = 10, (**d**) ground truth threshold = 6. (**e**, **f**) Evaluation on the second bootstrapped samples with a different random seed for sampling. (**e**) Comparison of AUCs of the precision-recall curves in (**b**) and (**d**). For both thresholds 6 and 10, FNA-Net yields significantly higher AUC than Faster R-CNN. (**f**) Comparison of maximum F1s at the optimal user thresholds found in (**a**) and (**c**). The performance of models on boostrapped samples at user thresholds 6 and 10 are shown in blue and orange, respectively. The statistical significance between models are shown. Significance was tested by the two-sided Wilcoxon signed-rank test. *p < 0.05. Error bars: one standard deviation.

Same as the previous section, the precision, recall, and F1 scores are calculated based on the number of TPs, FNs, and FPs (see Methods for details). When the user threshold which represents the minimum number of predicted follicular clusters, increases from 1 to 39 or to 23 (Fig. 5a, c), recall increases while precision decreases. In the case of the ground truth threshold, 10 (Fig. 5a), the classifier reached maximum F1, 0.918 with precision, 0.884, and recall, 0.955, when the user threshold is 26. Similarly, in the case of the ground truth threshold, 6 (Fig. 5c), the classifier reached maximum F1, 0.811 with precision, 0.720, and recall, 0.928, when the user threshold is 19. These results show that the user can adjust the user threshold to optimize the classifier performance by balancing the values of precision and recall. The user thresholds are much greater than the ground truth thresholds because FNA-Net tends to overestimate the number of follicular clusters in a slide.

High recall and low precision in the slide classification means that FNA-Net is careful not to classify inadequate slides to be adequate but sometimes even classify adequate slides to be inadequate. Clinically, having a high recall/low precision is preferred to having a low recall/high precision because it is more costly to have a high number of FNs than a high number of FPs. If the model incorrectly classified a slide to be adequate when it is inadequate (FN), the inadequacy of the slides would be found after staining the slide a few days later. Then the patient would have to revisit the clinic for the second FNA biopsy procedure. Therefore, giving more weight on recall than precision when choosing the user threshold can be desirable rather than maximizing F1 score.

To compare the performance of the three models, we bootstrapped another 10,000 samples from the test set using a different random seed to show consistency of our results regardless of which samples are bootstrapped. First, we plot precision-recall curves over a wide range of threshold values and calculate their AUC (Area Under the Curve) (Fig. 5b, d, e). We also applied the user threshold that maximized the F-1 scores in the previous samples (Fig. 5a, b) to the second bootstrapped samples to compare the model performance (Fig. 5f). When the ground truth threshold equals 10, FNA-Net has the highest AUC of 0.953 and F1 of 0.915, and MTL follows FNA-Net with an AUC of 0.941 and an F1 of 0.866. Faster R-CNN has an AUC of 0.849 and an F1 of 0.348 due to low precision over many threshold values. When the ground truth threshold equals 6, the overall performance of the models decreased because models overestimate the number of follicular clusters and passes some inadequate slides; an AUC of 0.840 and an F1 of 0.790 for FNA-Net, an AUC of 0.807 and an F1 of 0.693 for MTL, and an AUC of 0.711 and an F1 of 0.174 for Faster R-CNN. However, FNA-Net significantly achieves higher performance than standard Faster R-CNN, regardless of the ground truth thresholds (Fig. 5e, f) (*p*-value: 0.0156 for RCNN vs FNA-Net, Table S3, 4). This is because combining the outputs of the MTL classifier and Faster R-CNN increases the IOU and reduces the number of false positives in the follicular cluster detection.

## Discussion

We showed that our pipeline, FNA-Net, comprised of a low-cost imaging system and deep neural networks, can be used by clinicians at outpatient clinics to evaluate unstained thyroid FNA slides in situ and determine whether further aspirations are needed. High precision detection of follicular clusters by FNA-Net allowed us to build a simple classifier to screen inadequate slides by counting the number of identified follicular clusters. A user can flexibly adjust the recall and precision of the detection of inadequate slides depending on the costs of Type I/II errors. This will substantially reduce delays and costs in diagnosis and patient revisits related to FNA biopsy. Moreover, FNA-Net can be applied to other FNA samples in many benign or malignant diseases if different labels are provided for training.

For a proof of concept, the evaluation of slide screening was performed on simulated slides by bootstrapped resampling, due to the limited amount of test set. To deploy our model, we need to train our model with more extensive datasets and perform a prospective study to validate it in clinical settings. For more accurate follicular cluster detection, unstained images can be taken in higher magnification, such as 100X. At the current magnification of 20X, finding general regions of follicular clusters is possible, but distinguishing individual follicular cells are difficult. However, higher magnification will significantly increase the time required to acquire images of the slide, so the current magnification of 20X is the most appropriate for fast screening. So far, we have only utilized stained images for labeling the follicular, but we can employ the image-to-image translation method^31^ to virtually stain the unstained images and train FNA-Net on the stained images. By adding color information to the unstained image, FNA-Net may distinguish follicular clusters more easily.

FNA-Net demonstrated appealing deep learning applications to the point-of-care (POC) devices analyzing complex FNA samples. It did not require time-consuming manual staining processes and high-end light microscopes, and the deep learning inference can be made at the local device level. Therefore, we envision that FNA-Net embedded in POC devices can significantly reduce the diagnosis costs and improve the care of patients.

## Methods

### Dataset

FNA was performed on thyroid nodules using plastic syringes with 25- or 27-gauge needles. Two to four passes were performed, and the material was placed and smeared on positively charged slides. Then, the needles were rinsed in CytoRich Red vials to prepare a ThinPrep slide with the remaining material. The smear slides were immediately placed and kept in vials filled with 95% alcohol until they were transported to the cytology lab, where they were imaged. Then the slides were stained with Papanicolaou stain and reimaged (Fig. 2a). In total, unstained and stained FNA biopsy samples were taken from six patients, with a total of 21 slides to evaluate. All studies were performed in accordance with the guidelines and regulations of University of Massachusetts Medical School institutional review board (IRB) protocol, and approved by the IRB at University of Massachusetts Medical School (IRB ID: H00013974). Patient written consent was waived by the IRB at University of Massachusetts Medical School because the research was restricted to the analysis of de-identified cytology specimens.

A low-cost slide scanner attached to a bright-field microscope was used to take images of all FNA biopsy samples. The size of the glass slides was 75 by 26 mm and about 1 mm thick. Ground truth follicular clusters in unstained images were labeled with the help of two pathologists. The label has two categories: follicular cluster and background. Follicular clusters are colored white, and the background is colored black.

From 21 slides of unstained FNA biopsy samples from six patients, we obtained 287 images of size 2592 × 1944 pixels with three channels. Each unstained image was matched with its corresponding stained image to label the location of follicular clusters. On each unstained image, we labeled follicular clusters containing at least 10–15 cohesive follicular cells^2,29,30^. Images without any follicular clusters are classified as background class. The ratio of follicular cluster class to background class among 287 images is about 1:4. Images from unstained smears were taken at 20× magnification, and images from stained smears were taken at 100× magnification for accurate identification of follicular clusters and labeling.

### Slide scanner

Whole slide imaging is one of the ways to collect digital images for primary diagnosis, but it is not available in many pathology departments^32^. Therefore, we built a low-cost slide scanner that is more affordable than commercially available whole slide scanners to take digital images of the whole slide. The scanner moves a slide vertically and laterally on a flat surface via small knobs. The entire device fits in a footprint about 6 inches by 3 inches and stands less than 3 inches tall. Also, it is lightweight and can be placed on any microscope stage with a simple hand clamp (Fig. 2b, c**)**. OMAX mechanical stage was chosen as the foundation to develop automated capabilities. This device was automated by mounting two 26Ncm NEMA 17 motors next to the knobs and connecting them with large gears. A 12 V, 2Amp DC power supply was used to power the motors. The mounting frames and gears were all custom designed, and 3D printed to fit desired constraints. An Arduino CNC shield was used as the motor controller because it was very small, inexpensive, and could operate through a serial port interface. The Arduino mounts directly to the device on the clamping platform. Arduino in LSS is interfaced with an Amscope USB camera through the serial port connection. The script written in Python sends G-code commands to incrementally step through all locations on a slide, taking pictures at each step (Fig. 2d). LSS takes images of the entire slide surface in 3 min at 20× magnification.

### Dataset preparation

Images from six patients were combined, randomly shuffled, and split into 7 smaller groups, each group containing 33 images in background class and 8 images in follicular class. One of the groups is left out as a test set, or validation set, and the remaining 5 groups were used for training (Table 2). To train deep learning models, images of size 2566 × 1966 pixels were cropped into 256 × 256 patches with 50% overlap with neighboring patches. Overlapping has the effect of data augmentation and preventing small follicular clusters from being ignored when the follicular cluster area is split into two patches during cropping. Given ground truth labels for follicular clusters, we considered the patch to contain follicular clusters if the ground truth follicular cluster’s area was greater than 655 (256 × 256 × 1%) pixels in the patch.

**Table 2:** Split dataset into train, validation, and test sets.

	Follicular	Negative	Total
Training set	40	165	205
Validation set	8	33	41
Test set	8	33	41
Total	56	231	287

### Multi-task learning for patch-wise classification

Classification loss is the sigmoid focal loss to reduce the effect of too many negative examples in the imbalanced dataset. Regression loss is the mean absolute error (MAE). Autoencoder, or image reconstruction loss, is mean squared error (MSE). The segmentation loss is binary cross-entropy loss used for segmentation by U-Net^27^. In the equations below, y_i_ represents the ground truth value and ŷ_i_ represents the predicted value by the model. β and γ are equal to 0.5 and 2, respectively. In the total loss, α is the trainable parameter and is first initialized to 0 such that each loss starts with equal weights. The final three terms are for regularization to prevent each loss weight from converging to 0. We didn’t put any trainable weight for the classification task because we empirically observed that the accuracy of the model increases without trainable loss weight for the classification task.

$$L_{cls} = - \frac{1}{n} \mathop \sum \limits_{i = 1}^{n} \beta \left( {1 - \hat{y}_{i} } \right)^{\gamma } \log \hat{y}_{i}$$

$$L_{reg} = - \frac{1}{n} \mathop \sum \limits_{i = 1}^{n} \left| {y_{i} - \hat{y}_{i} } \right|$$

$$L_{aut} = - \frac{1}{n} \mathop \sum \limits_{i = 1}^{n} (y_{i} - \hat{y}_{i} )^{2}$$

$$L_{seg} = - \frac{1}{n} \mathop \sum \limits_{i = 1}^{n} (y_i \log \hat{y}_{i} + (1-y_i) \log(1-\hat{y_i}))$$

$$L_{total} = L_{cls} + e^{{\alpha_{1} }} L_{reg} + e^{{\alpha_{2} }} L_{aut} + e^{{\alpha_{3} }} L_{seg} + \alpha_{1} + \alpha_{2} + \alpha_{3} .$$

### Object detection algorithm

Instead of pixel-wise classification in semantic segmentation, object detection draws a rectangular box around the object which the model was trained to detect. Since our goal is to screen the adequacy of the FNA biopsy sample, accurate segmentation of the follicular clusters is not necessary. Instead, counting follicular clusters per image suffices the need. Among various object detection models, some have a fast inference time, such as YOLO^33^, while others have higher accuracy with slow inference time. Our criterion for choosing the model is the high accuracy with a total inference time of less than several seconds. Using Tensorflow Object Detection API^34^, we trained Faster R-CNN with Inception-Resnet v2^35^ backbone and atrous convolutions on our training/validation dataset.

Faster R-CNN is the two-stage object detection algorithm. In the first stage, features are extracted by the backbone CNN and several regions of interest are selected by the region proposal network using extracted features. In the second stage, proposed regions are transformed to have the same size through the ROI pooling layer, and multiple fully-connected (FC) layers are used to perform multiple tasks such as classifying regions and estimating the location of each region to draw a boundary box around.

### Training details

The MTL classifiers were configured with the following hyperparameters: Adam optimizer with learning rate = 10^–5^, batch size = 64, input size = 128, output size = 68, early stopping patience = 10. VGG19 was ImageNet pretrained and fine-tuned without freezing any weights. The binary cross-entropy was used as a loss function for training. To avoid overfitting, we used the early stopping, so training stopped when the validation loss did not decrease during the three consecutive epochs. Early stopping patience was 3, and the maximum epoch was 100. Early stopping ends the training when there is a sign of overfitting, which decreases training loss when validation loss is increasing. For all other parameters, default values in the Tensorflow library were used. MARS-Net^23,36^ was used to train MTL classifiers. For Faster R-CNN with Inception-Resnet v2^35^ backbone and atrous convolutions, changing the default hyperparameters provided by the Tensorflow Object Detection API^34^ did not improve its performance so default hyper parameters were used except for decreasing the number of output classes to 2 from 90. All models were trained by TensorFlow v2.4 and RTX Titan GPU, and CUDA v11.3.

### Evaluation of follicular cluster detection

IOU is calculated by dividing the intersection between the ground truth follicular cluster and the predicted bounding box by the union of the ground truth follicular cluster and the predicted bounding box. Then, the IOU from each image is used to calculate an average IOU. The image is not used in the calculation of IOU if the image does not contain any ground truth follicular cluster and the model does not make any false positive predictions on that image.

For evaluation of follicular cluster detection, we considered the overlap between the predicted box and the ground truth box to be a true positive if the overlapped area contains at least one pixel. This is because the highest F1 score, precision and recall happen at the one pixel overlap threshold, as shown in Supplementary Fig. 2. Since our goal is to count the number of follicular clusters in the image, instead of precisely locating the follicular clusters, we used one pixel overlap threshold for evaluation. When there are multiple predicted box nearby one ground truth box, only one of predicted boxes is counted as a true positive and the other predicted boxes are ignored. If the region has a ground truth box without a predicted box, it is a false negative. If the region does not have a ground truth box but has a predicted box, it is a false positive. We did not count true negatives since true negative is not necessary to calculate precision, recall, and F1 score.

From the follicular cluster detection predicted by the trained models, we counted True Positive (TP), True Negative (TN), False Negative (FN), and False Positive (FP). TP means that the ground truth follicular cluster is correctly classified as a follicular cluster. TN means that the ground truth background class is correctly classified as the background, not the follicular cluster. FN means predicting a region to be a background location when that region has a follicular cluster. FP represents predicting a region to be a follicular cluster when that region is a background. Using TP, TN, FN, and FP, we calculated Precision, Recall, and F1 score with the following formulas.

$$IOU = Intersection/Union$$

$$Precision = TP/\left( {TP + FP} \right)$$

$$Recall = TP/\left( {TP + FN} \right)$$

$$F1 = 2*Precision*Recall/\left( {Precision + Recall} \right).$$

### 7-Fold cross validation for evaluation

To compare performance of models reliably, we used seven fold cross-validation for evaluation of MTL classifiers, Faster R-CNN, FNA-Net on follicular detection per image and on bootstrapped samples. In seven fold cross validation, the dataset is split into 7 folds without redundant data. Among 7 folds, 5 of them are used as the training set, one of them is used for the validation set and the last one of them is used for the test set. In total, there are seven different combinations of training, validation and test sets. The same neural network is trained on seven different combinations of training sets to produce seven different trained models. Each of trained models are evaluated on the different test sets, which yields seven values for each of the model performances such as IOU, precision, recall and F1 score. We computed average and standard deviation from those seven values and assessed the significance of model performance differences by the two-sided Wilcoxon signed-rank test, which does not assume Gaussian distribution.

### Supplementary Information

Supplementary Figures.

Supplementary Tables.

## Data Availability

The datasets used in the current study are available from the corresponding author on a reasonable request.

## Code availability

The code used in the current study is available at Github repository (https://github.com/kleelab-bch/FNA).
