# Author Correction: Screening adequacy of unstained thyroid fine needle aspiration samples using a deep learning-based classifier

**DOI:** 10.1038/s41598-023-42267-y

**Published:** 2023-09-12

**Authors:** Junbong Jang, Young H. Kim, Brian Westgate, Yang Zong, Caleb Hallinan, Ali Akalin, Kwonmoo Lee

**Affiliations:** 1 Department of Biomedical Engineering, Worcester Polytechnic Institute, Worcester, MA 01609 USA; 2 Vascular Biology Program, Boston Children’s Hospital, Boston, MA 02115 USA; 3 Department of Radiology, University of Massachusetts Medical School, Worcester, MA 01655 USA; 4 Department of Pathology, University of Massachusetts Medical School, Worcester, MA 01655 USA; 5 Department of Surgery, Harvard Medical School, Boston, MA 02115 USA

Correction to: *Scientific Reports* 10.1038/s41598-023-40652-1, published online 19 August 2023

The original version of this Article contained an error in the spelling of the author Young H. Kim which was incorrectly given as Young Kim.

The original Article has been corrected.

