# Supplementary material for: Screening adequacy of unstained thyroid fine needle aspiration samples using a deep learning-based classifier
Source: Sci Rep. 2023 Aug 19;13:13525. doi: 10.1038/s41598-023-40652-1 (PMC10439921; doi:10.1038/s41598-023-40652-1)
Supplement: Supplementary file 1 — Supplementary Figures. [file 41598_2023_40652_MOESM1_ESM.pdf]

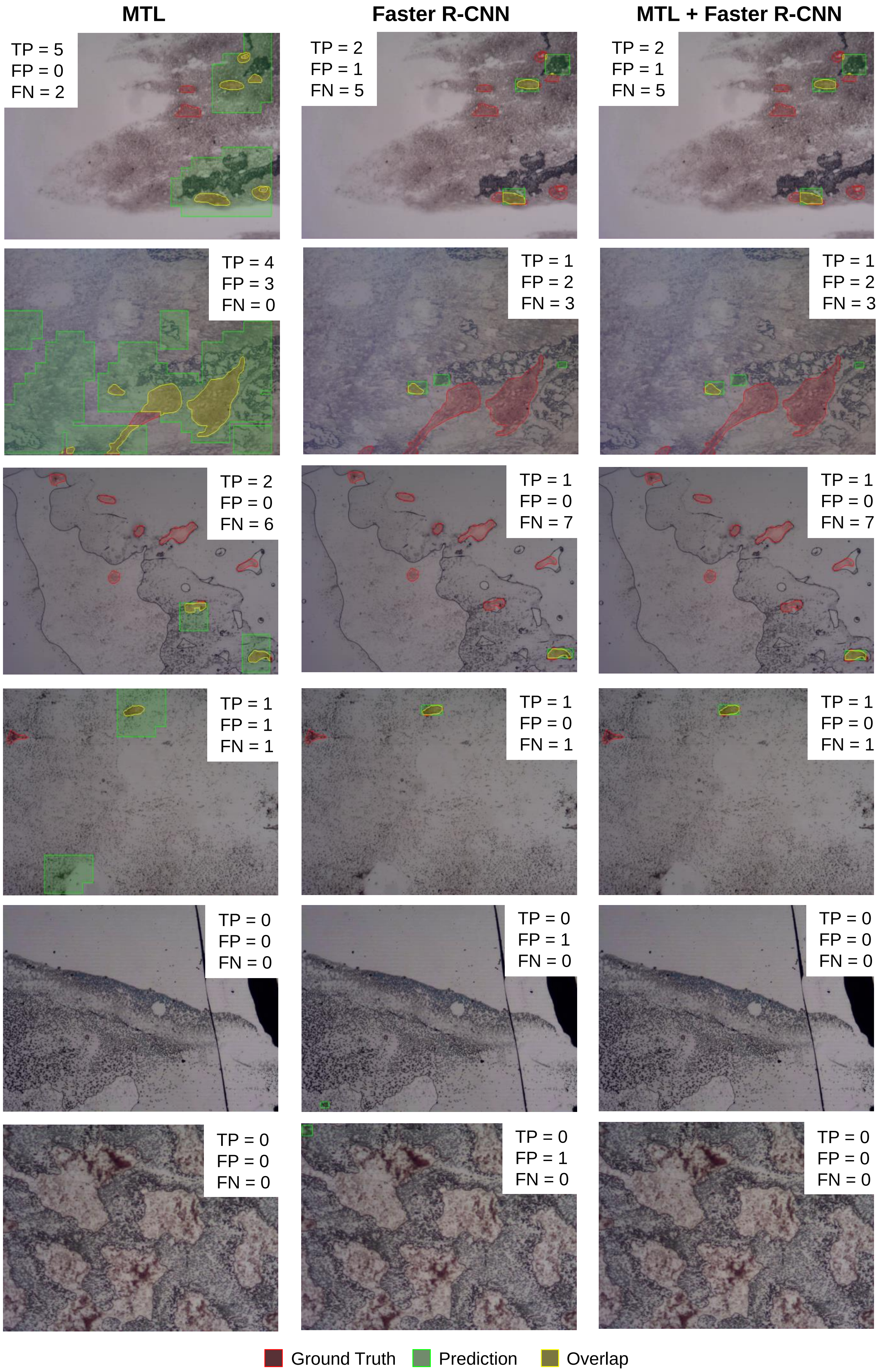

MTL
Faster R-CNN
MTL + Faster R-CNN
TP = 5
FP = 0
FN = 2
TP = 2
FP = 1
FN = 5
TP = 2
FP = 1
FN = 5
TP = 4
FP = 3
FN = 0
TP = 1
FP = 2
FN = 3
TP = 1
FP = 2
FN = 3
TP = 2
FP = 0
FN = 6
TP = 1
FP = 0
FN = 7
TP = 1
FP = 0
FN = 7
TP = 1
FP = 1
FN = 1
TP = 1
FP = 0
FN = 1
TP = 1
FP = 0
FN = 1
TP = 0
FP = 0
FN = 0
TP = 0
FP = 1
FN = 0
TP = 0
FP = 0
FN = 0
TP = 0
FP = 0
FN = 0
TP = 0
FP = 1
FN = 0
TP = 0
FP = 0
FN = 0
Ground Truth
Prediction
Overlap

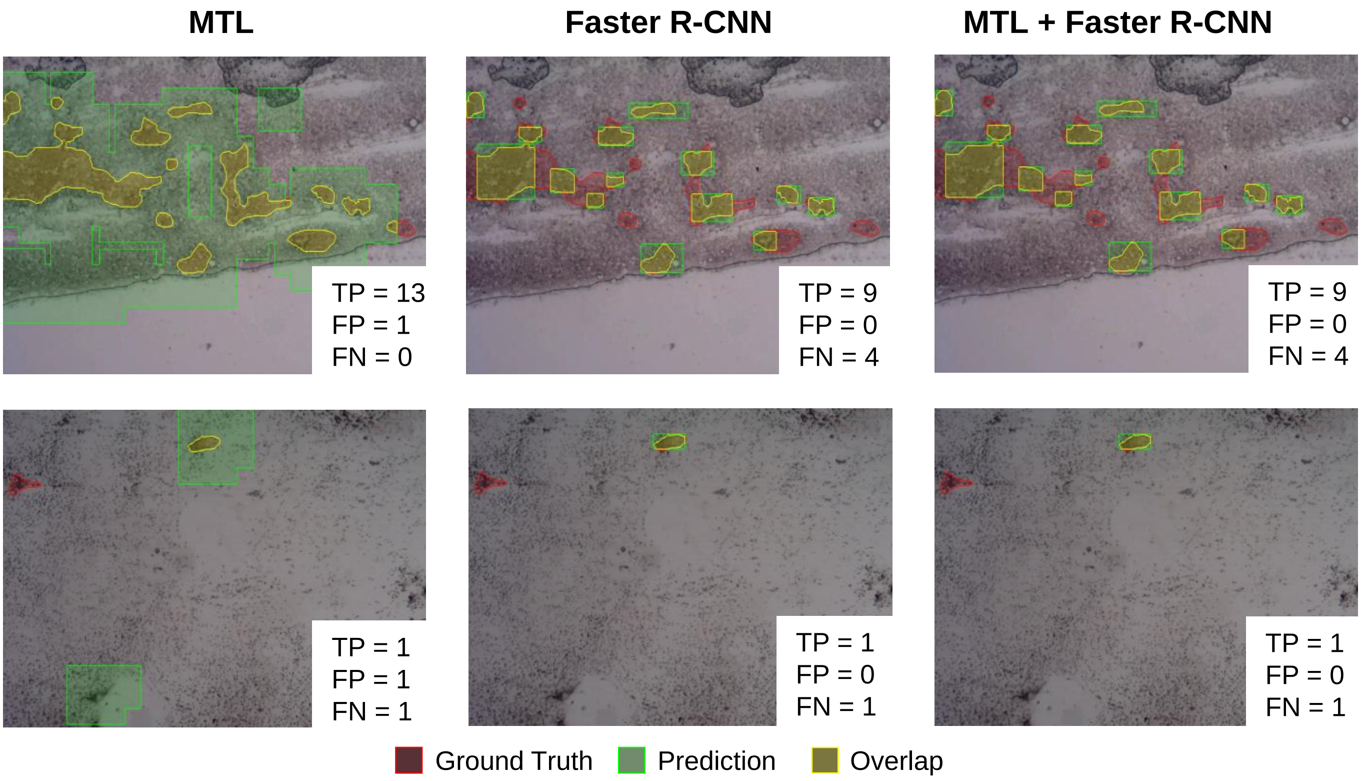

Supplementary Figure 1. Comparison of follicular cluster detection by MTL, Faster R-CNN and FNA-Net, related to Figure 3. The first column is the follicular cluster detection by the MTL model. The second column is the follicular cluster detection by the Faster R-CNN model. The third or last column is the follicular cluster detection by combing predictions from MTL and Faster R-CNN models. In the corner of each image, the number of true positives, false positives and false negatives are indicated next to the letters TP, FP, and FN, respectively. Each row has the same underlying image with the same ground truth. The ground truth is colored red, detection by the model is colored green, and the overlap between ground truth and detection is colored yellow.

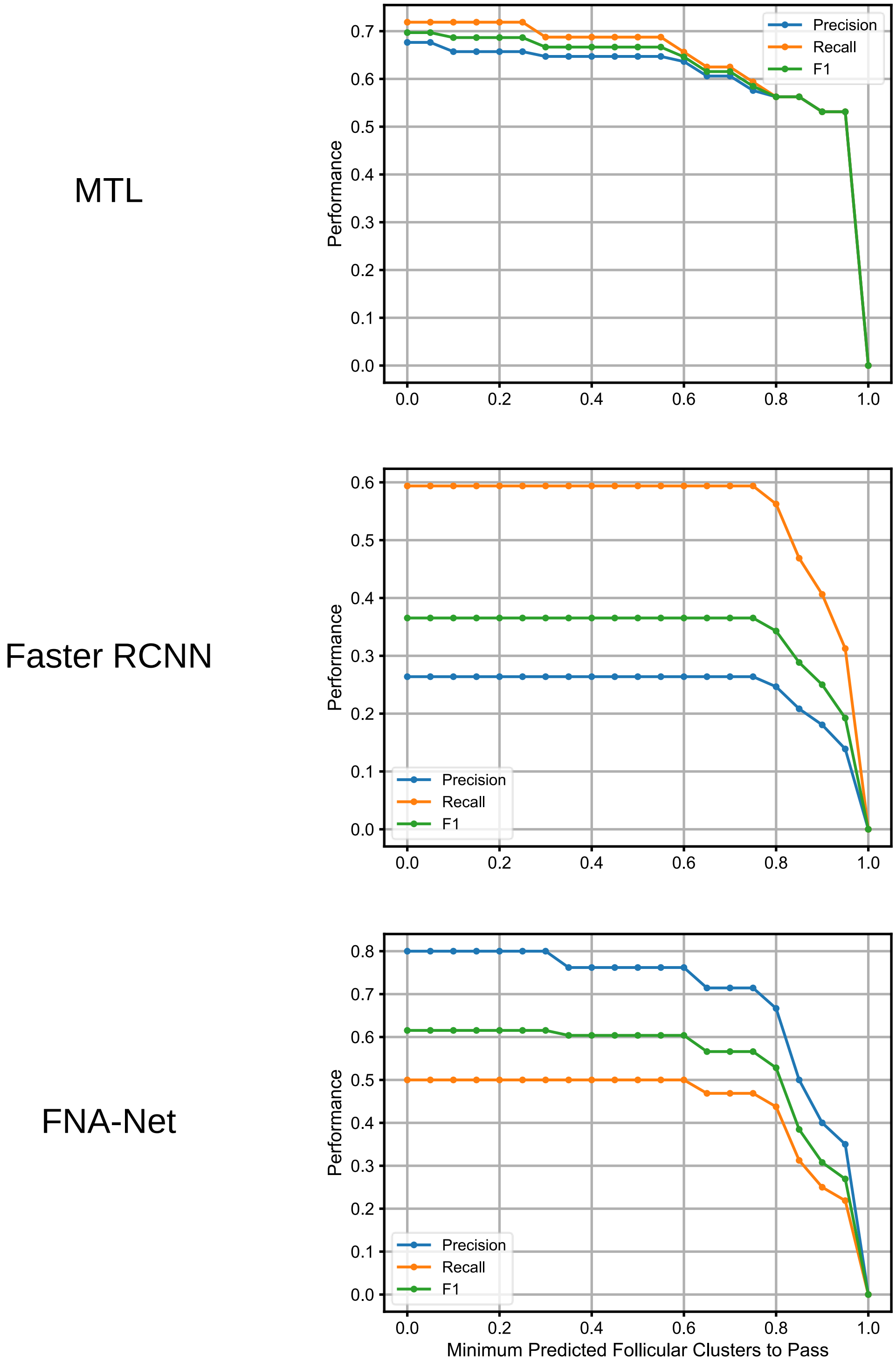

Supplementary Figure 2. Effect of changing overlap threshold for detection status. Overlap threshold varies from 1 pixel (0%) to 100%. The first, second, and third row represents the follicular cluster detection by the MTL model, the Faster R-CNN and FNA-Net, respectively. Performance in one of the fold in the 7-fold cross validation is shown but the trend that the performance increases as overlap threshold decreases is the same for all other folds.
