# Supplementary material for: Screening adequacy of unstained thyroid fine needle aspiration samples using a deep learning-based classifier
Source: Sci Rep. 2023 Aug 19;13:13525. doi: 10.1038/s41598-023-40652-1 (PMC10439921; doi:10.1038/s41598-023-40652-1)
Supplement: Supplementary file 2 — Supplementary Tables. [file 41598_2023_40652_MOESM2_ESM.pdf]

Supplementary Information: Statistical Testing (P-values)

Supplementary Table S1. P-values (**Fig. 3a**) of model comparisons for different performance measures by two-sided Wilcoxon signed-rank test.

Mode Comparison	Precision	Recall	F1	IOU
6 vs. 4	0.219	0.686	0.031	0.016
6 vs. 3	0.156	0.016	0.938	0.031
6 vs. 2	0.109	0.249	0.813	0.031
6 vs. 5	0.297	0.375	0.813	0.156
6 vs. 7	0.297	0.893	0.688	0.297
6 vs. 1	0.031	0.345	0.078	0.016
4 vs. 3	0.688	0.028	0.469	0.297
4 vs. 2	0.813	0.249	0.938	0.156
4 vs. 5	0.688	0.463	0.297	0.047
4 vs. 7	0.047	0.813	0.016	0.016
4 vs. 1	1.000	0.345	0.375	0.578
3 vs. 2	0.813	0.500	0.688	0.578
3 vs. 5	0.219	0.116	0.938	0.297
3 vs. 7	0.047	0.028	0.938	0.031
3 vs. 1	0.469	0.068	0.109	0.688
2 vs. 5	0.109	0.345	0.938	0.078
2 vs. 7	0.016	0.075	0.938	0.078
2 vs. 1	0.469	0.156	0.297	0.813
5 vs. 7	0.078	0.144	0.688	0.047
5 vs. 1	0.297	0.116	0.219	0.375
7 vs. 1	0.016	0.753	0.078	0.016

Supplementary Table S2. P-values (**Fig. 3c**) of model comparisons for different performance measures by two-sided Wilcoxon signed-rank test.

Mode Comparison	Precision	Recall	F1	IOU
MTL vs. Faster R-CNN	0.0469	0.6002	0.0313	0.0156
MTL vs. FNA-Net	0.2969	0.0277	0.0313	0.0156
Faster R-CNN vs. FNA-Net	0.0156	0.0156	0.2969	0.1563

Supplementary Table S3. P-values (**Fig. 5e**) of AUC comparisons by two-sided Wilcoxon signed-rank test.

Threshold	Mode Comparison	Precision
6	MTL vs RCNN	0.078
6	RCNN vs FNA-Net	0.016
6	MTL vs FNA-Net	0.578
10	MTL vs RCNN	0.219
10	RCNN vs FNA-Net	0.047

10	MTL vs FNA-Net	0.375

Supplementary Table S4. P-values (**Fig. 5f**.) of F1 comparisons by two-sided Wilcoxon signed-rank test.

Threshold	Mode Comparison	Precision
6	MTL vs RCNN	0.0313
6	RCNN vs FNA-Net	0.0156
6	MTL vs FNA-Net	0.1563
10	MTL vs RCNN	0.0313
10	RCNN vs FNA-Net	0.0156
10	MTL vs FNA-Net	0.1094
